# Methyltransferase Inhibitors: Competing with, or Exploiting the Bound Cofactor

**DOI:** 10.3390/molecules24244492

**Published:** 2019-12-08

**Authors:** Renato Ferreira de Freitas, Danton Ivanochko, Matthieu Schapira

**Affiliations:** 1 Centro de Ciências Naturais e Humanas, Universidade Federal do ABC, Rua Arcturus 3, São Bernardo do Campo, SP 09606-070, Brazil; 2 Structural Genomics Consortium, University of Toronto, MaRS Centre, South Tower, 101 College St., Suite 700, Toronto, ON M5G 1L7, Canada; 3 Princess Margaret Cancer Centre and Department of Medical Biophysics, University of Toronto, Toronto, ON M5G 2M9, Canada; 4 Department of Pharmacology and Toxicology, University of Toronto, 1 King’s College Circle, Toronto, ON M5S 1A8, Canada

**Keywords:** methyltransferases, S-adenosylmethionine, inhibitors, mechanism of action

## Abstract

Protein methyltransferases (PMTs) are enzymes involved in epigenetic mechanisms, DNA repair, and other cellular machineries critical to cellular identity and function, and are an important target class in chemical biology and drug discovery. Central to the enzymatic reaction is the transfer of a methyl group from the cofactor S-adenosylmethionine (SAM) to a substrate protein. Here we review how the essentiality of SAM for catalysis is exploited by chemical inhibitors. Occupying the cofactor binding pocket to compete with SAM can be hindered by the hydrophilic nature of this site, but structural studies of compounds now in the clinic revealed that inhibitors could either occupy juxtaposed pockets to overlap minimally, but sufficiently with the bound cofactor, or induce large conformational remodeling leading to a more druggable binding site. Rather than competing with the cofactor, other inhibitors compete with the substrate and rely on bound SAM, either to allosterically stabilize the substrate binding site, or for direct SAM-inhibitor interactions.

## 1. Introduction

Protein methyltransferases (PMTs) transfer a methyl group from the cofactor S-adenosylmethionine (SAM) to a substrate protein. In humans, 50 SET domain methyltransferases methylate lysine side-chains, and 13 Rossman fold (also known as Class I) enzymes methylate arginine or lysine side-chains [1]. Protein substrates are involved in a diverse array of signaling machineries, including epigenetic mechanisms, DNA damage response, and RNA processing. A number of PMTs are considered attractive therapeutic targets [2,3,4]: EZH2, the catalytic subunit of the polycomb repressive complex 2 (PRC2), tri-methylates lysine 27 of histone 3 (H3K27), and oncogenic hyper-trimethylation of H3K27 is driven by overexpression or activating mutations of EZH2 in lymphoma. EZH2 inhibitors, now in clinical trial, are showing promising anticancer effect in lymphoma, as well as in solid tumors carrying mutations in the SWI/SNF chromatin remodeling complex [5]. Protein arginine methyltransferase 5 (PRMT5) methylates histones and components of the survival of motor neurons (SMN) complex, involved in RNA splicing. While the exact biology of PRMT5 inhibitors remains unclear, advanced compounds are now in clinical trial against lymphoma and solid tumors [6]. Drugs targeting the PMTs DOT1L and PRMT1, as well as EED (an obligatory binding partner of the PMT MLL1), are also in various phases of clinical trial. Potent inhibitors have been reported for both SET domain (EZH2, EHMT1/2, SMYD2, SMYD3, SUV420H1/2, SETD7) and Class I (DOT1L, PRMT5, PRMT1/8, PRMT3, PRMT4, PRMT6) enzymes [1,7], but some PMTs with strong disease association remain so far without high-quality drug-like ligands: recurrent chromosomal aberrations affecting the H3K36 methyltransferase NSD2 lead to oncogenic hyper-trimethylation of H3K36 in myeloma and leukemia [8,9], while amplification of the H3K9 methyltransferase SETDB1 is a driving factor in lung tumorigenesis and melanoma [10,11].

**Figure 1:**
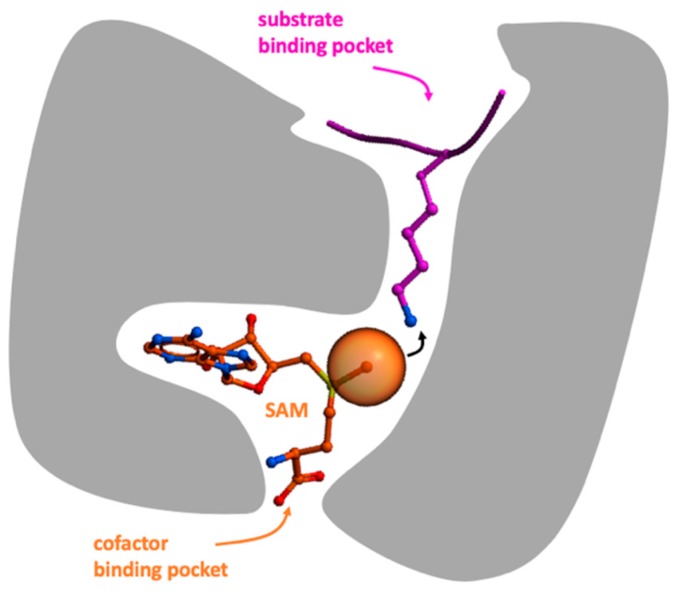
Protein methyltransferases (PMTs) (grey) catalyze the transfer of a methyl group (orange ball) from the cofactor S-adenosylmethionine (SAM) (orange) to a substrate peptide (pink) that occupies a distinct binding pocket.

Most PMT inhibitors directly compete with either the cofactor or the substrate, each occupying juxtaposed but distinct binding pockets (Figure 1). SAM binds in one of two conserved binding poses, but the structural diversity of the cofactor binding site is nevertheless sufficient for the development of selective inhibitors [12]. More challenging for drug discovery is the fact that SAM is a highly hydrophilic molecule, and competitors must be sufficiently polar to occupy the SAM binding pocket, but sufficiently hydrophobic to cross cell membranes. Additionally, compounds must compete with high cellular levels of SAM. In comparison, the substrate binding site, which relies on a high structural diversity for selective substrate recruitment and is not excessively polar, seems to be chemically more tractable. Nevertheless, the first compounds to reach the clinic (DOT1L and EZH2 inhibitors) both acted as SAM competitors [13,14,15], and structural studies revealed different mechanisms used by these compounds to overcome the hydrophilic nature of the cofactor binding site. Later studies showed that some substrate-competing molecules, such as clinical inhibitors of PRMT5, were engaged in direct and critical interactions with the bound cofactor to potently inhibit their target [16].

Here, we review the structural chemistry of methyltransferase inhibitors, focusing first on cofactor-competing compounds and next on cofactor-dependent substrate competitors.

## 2. Cofactor Competitors

**Figure 2:**
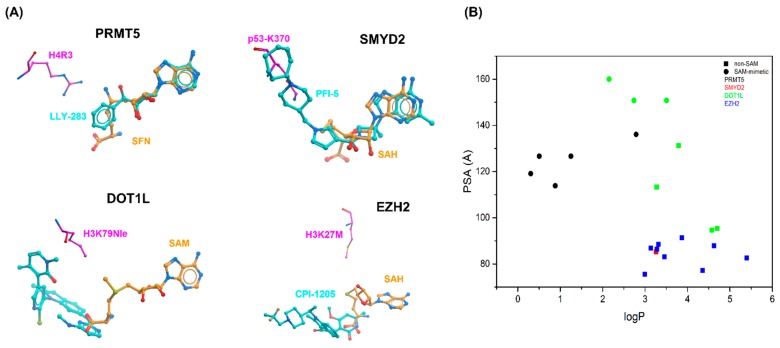
SAM competitors vary in their binding mode and physico-chemical properties. (**A**) Binding mode of inhibitors relative to the position of the cofactor and substrate lysine or arginine. (top-left) PRMT5: Sinefungin (orange) + H4R3 (magenta) (PDB ID: 4GQB) and LLY283 (cyan, PDB ID: 6CKC). (top-right) SMYD2: SAH (orange) + p53-K370 (magenta) (PDB ID: 3TG5) and PFI-5 (cyan, PDB ID: 6CBY). (bottom-left) DOT1L: SAM (orange) + K3K79 (magenta) (PDB ID: 6NJ9) and an inhibitor (PDB ID: 5DSX). (bottom-right) EZH2: SAH (orange) + H3K27M (magenta) (PDB ID: 5HYN) and CPI-1205 (cyan, 5LS6). (**B**) Physico-chemical properties: clogP and PSA are plotted for SAM mimetics (circles) and compounds chemically distant from SAM (squares). Compounds are colour-coded according to their protein targets. (The SMYD2 inhibitor PFI-5 contains an adenine ring that is conserved in SAM, but this represents only about one third of the compound, which is therefore not classified as SAM mimetic).

Compounds that directly compete with the cofactor can be close SAM mimetics, such as the PRMT5 inhibitor LLY283 [17], may only retain the adenine ring of SAM (ex: SMYD2 inhibitor PFI-5) [18], or are chemically unrelated to the cofactor (ex: EZH2 and second generation DOT1L inhibitors). While cofactor mimetics overlap extensively with SAM, structurally distinct compounds may exploit binding pockets adjacent to the cofactor and overlap only minimally—but still sufficiently—with SAM (Figure 2A). To be cell permeable, SAM analogs must evolve towards an area of the chemical space with higher logP and lower polar surface area (PSA). Indeed, the calculated logP and PSA values of SAM are −5.8 and 182.6 Å^2^, respectively (calculated with ChemAxon), and SAM (as well as the pan-methyltransferase inhibitor sinefungin) show poor cell membrane permeability. With this in mind, all chemical probes and clinical candidates that will be discussed in this section were designed to achieve higher logP and lower PSA values. Expectedly, SAM-mimetics have higher PSA than compounds with structures distinct from the cofactor (Figure 2B).

SAM-competitive inhibitors have to compete with high intracellular concentration of cofactor, and the relative affinity (*K*_M_) of SAM for PMTs can in some cases be an almost insurmountable challenge. For instance, while the *K*_M_ of SAM for EZH2 is modest (low µM), it is in the low nM for SMYD2 [19,20]. To avoid large shifts in potency from biochemical to cellular assays [21] this key point should be considered in the optimization of inhibitors, as discussed below for SMYD2-targeting compounds. Some of the closest analogs of SAM that display target selectivity are PRMT5 inhibitors, which we will review first.

### 2.1. PRMT5

PRMT5 is a type II protein arginine methyltransferase (PRMT) that catalyzes the formation of symmetrical dimethylarginine (sDMA) on histones and non-histone substrates. There is evidence that PRMT5 plays important roles in various cellular processes, including gene expression and RNA splicing [22,23,24,25,26]. PRMT5 is a promising cancer target in several malignancies including lymphoma and glioblastoma [6,16,27,28], and PRMT5 inhibitors are in clinical trial against lymphomas and solid tumors.

Some of the first SAM-competitive PRMT5 inhibitors reported in the literature include close analogs of the cofactor or compounds designed to occupy both the cofactor and the arginine binding site [29,30]. However, these inhibitors lacked sufficient selectivity or cellular activity to be used as chemical probes. Despite its small size (molecular weight MW = 297 Da), the endogenous ligand methylthioadenosine (MTA) (3) is a potent and selective PRMT5 inhibitor (IC_50_ = 260 nM) and represented the first useful starting point to develop SAM-mimetic inhibitors of PRMT5 (Figure 2A) [31,32,33].

**Figure 3:**
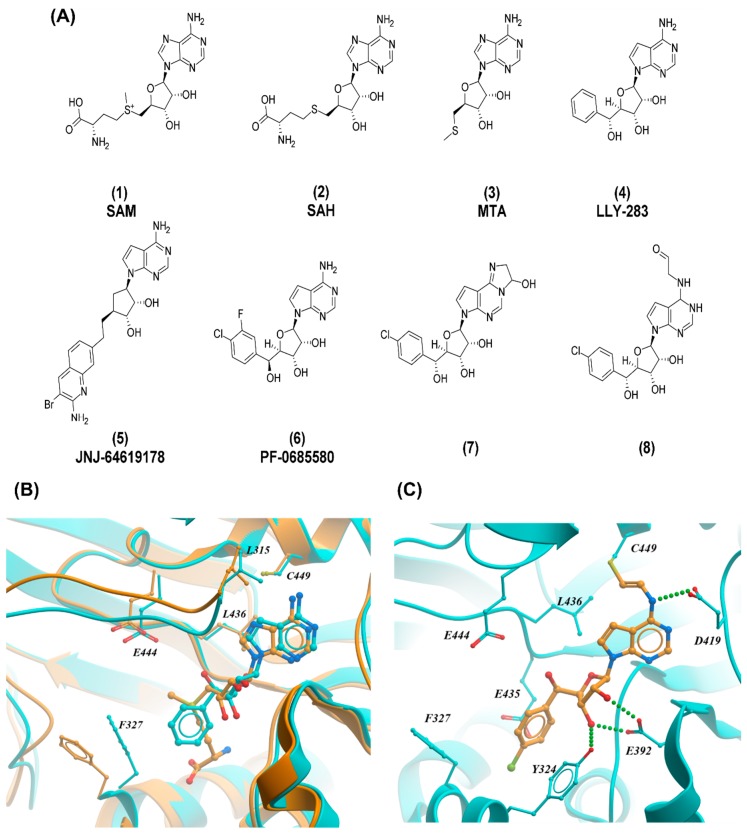
SAM-competitive inhibition of PRMT5. (**A**) Structure of the cofactor SAM, the product SAH, and representative PRMT5 inhibitors. (**B**) Crystal structure of PRMT5 in complex with SAM (orange; PDB ID: 4 × 61) and LLY-283 (cyan; PDB ID: 6CKC). (**C**) Crystal structure of the covalent inhibitor 8 in complex with PRMT5 (PDB ID: 6K1S). Hydrogen bonds are shown as green dotted lines.

The discovery of these prototype compounds as efficient SAM-competitive inhibitors of PRMT5 led to more potent molecules. LLY-283 (4) [17], JNJ-64619178 (5) [34], and PF-06939999 (structure not disclosed) are the first compounds to show potency, selectivity, and cellular activity (Figure 3A). The last two have entered clinical trials for the treatment of advanced solid tumors (Clinicaltrials.gov ID: NCT03573310 and NCT03854227).

LLY-283 inhibited PRMT5 enzymatic activity in vitro and in cells with an IC_50_ of 22 nM and 25 nM, respectively [17]. This inhibitor was highly selective for PRMT5 over a panel of 32 methyltransferases. It also showed antitumor activity in mouse xenografts when dosed orally. The biochemical and cellular potency could be increased through substitutions on the phenyl ring of LLY-283, which led to the discovery of compound PF-06855800 (6). This ligand includes a 4-chloro-3-fluorophenyl ring and has an inhibition constant *K*_i_ of 0.02 nM and a cell IC_50_ of 1.4 nM. The crystal structure of PRMT5 in complex with LLY-283 confirmed that the inhibitor binds at the cofactor binding site (Figure 2B). The binding pose of the adenine and ribose moieties is absolutely conserved with that observed in SAM.

The clinical candidate JNJ-64619178 is a potent (IC_50_ = 0.13 nM; EC_50_ = 0.25 nM) and selective PRMT5 inhibitor with favorable pharmacokinetics and safety properties. To rationalize the basis for the extreme potency of this inhibitor, a recent publication reports the modeling of the complex between PRMT5 and an analog of JNJ-64619178 [35]. According to this model, the inhibitor occupies the SAM binding pocket and extends into the substrate arginine binding pocket in a pseudo-irreversible binding mode with long residence time and extended pharmacodynamics (PD) effects.

A common feature shared by LLY-283, JNJ-64619178, and PF-06855800 is a deazapurine ring, which improves physico-chemical properties. As can be seen in Figure 3B, the nitrogen N7can be removed from the adenine ring without compromising the potency, since this atom is making only hydrophobic interactions with the receptor.

Cysteine 449 of PRMT5 is lining the cofactor binding site (Figure 3B) and is amenable to covalent targeting by small-molecules. The absence of this cysteine in other PRMTs should enable selective covalent inhibition. Proof-of-concept covalent PRMT5 inhibitors were recently reported [36]. These acrylamide derivatives of MTA were weak, but showed evidence of covalent engagement of wild type PRMT5 but not the mutant C449S. The same group reported a series of novel inhibitors that can react with C449 to form covalent adducts [37]. The hemiaminal 7 and aldehyde 8 inhibited PRMT5 with IC_50_ values of 11 nM and 19.5 nM, respectively. Both 7 and 8 showed dose-response inhibition of sDMA in cells with IC_50_ values of 12 and 22 nM, respectively, and 7 was completely selective for PRMT5 over PRMT1 and PRMT4. The crystal structure of PRMT5 in complex with 8 confirmed the existence of a covalent bond with the inhibitor (Figure 3C).

There have been some attempts to identify compounds that differ structurally from previously published SAM-based inhibitors using virtual screening [38,39,40]. One group provided experimental evidence for this mechanism of inhibition [39], but these inhibitors were less potent than the SAM-analogs.

### 2.2. SMYD2

SET and MYND domain-containing protein 2 (SMYD2) is a lysine methyltransferase which mediates the methylation of histones [41] and non-histones substrates such as p53, Rb, HSP90, and estrogen receptor α (ERα) [42,43,44,45]. Small molecule chemical probes targeting the binding site of the lysine substrate are helping to dissect the biology of SMYD2 [46,47,48,49]. However, until recently, there was no SMYD2 chemical probe exploiting the SAM-binding pocket.

The first SAM-competitive inhibitor of SMYD2 was discovered through the screening of a focused library mimicking the cofactor but with improved druglike properties [18]. The optimization of a promising hit resulted in compounds with low nanomolar IC_50_ values. Despite their high potency, initial compounds showed no effect on p53 methylation in MCF7 cells. This lack of cellular activity was attributed to the ultra-high affinity of SAM for SMYD2 (*K*_M_~70 nM) and the high intracellular concentration of the cofactor.

**Figure 4:**
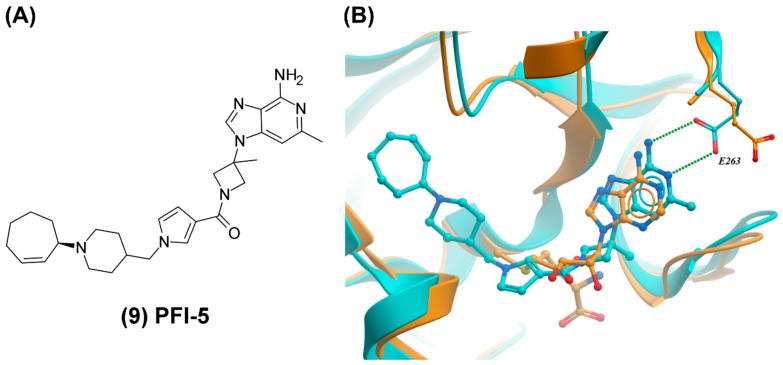
SAM-competitive inhibition of SET and MYND domain-containing protein 2 (SMYD2). (**A**) Structure of PFI-5. (**B**) Crystal structure of SMYD2 in complex with PFI-5 (cyan, PDB ID: 6CBY) and SAM (orange, PDB ID: 3TG4). While E263 is pointing towards the solvent in the SAM-bound structure, this residue forms a hydrogen (green dotted line) bond with the 3-deaza-adenine ring of PFI-5.

Further optimization and SAR studies conducted at elevated SAM concentrations to better model kinetics observed in cells resulted in the discovery of PFI-5 (9) which behaved as a potent SMYD2 inhibitor both in the presence of 70nM and 20 µM SAM (IC_50_ of 8 and 16 nM, respectively) (Figure 4A). This compound features a 3-deaza-adenine ring while the ribose and amino acid side chain of SAM are replaced with an azetidine group and a linear succession of three ring systems, respectively. PFI-5 inhibits methylation of p53 in MCF7 cells with an IC_50_ of 1.3 μM. Additionally, PFI-5 shows less than 20% inhibition against a panel of 33 protein lysine methyltransferases (PKMTs), PRMTs, DNMTs, and RNMTs when tested at 50 μM. The PKMTs SUV420H1 and SUV420H2 were the only enzymes that showed partial inhibition in the presence of 1 μM of PFI-5. In a cellular assay, the selectivity of PFI-5 for SMYD2 over SUV420H1 and SUV420H2 was around 30-fold.

The crystal structure of PFI-5 in complex with SMYD2 shows that the deaza-adenine ring occupies the SAM binding pocket (Figure 4B). The other end of the inhibitor deviates from the amino acid side chain of SAM and projects into the substrate lysine binding pocket (Figure 2A). The improved cellular activity of PFI-5 compared with its precursors is due both to increased lipophilicity and higher affinity.

### 2.3. DOT1L

DOT1L is the only known histone H3 lysine 79 (H3K79) methyltransferase [50]. H3K79 methylation plays a key role in gene transcription, DNA repair, and cell cycle regulation [51,52,53]. DOT1L is associated with the development and maintenance of MLL-rearranged leukemias and potent inhibitors have been reported [13,54,55,56].

**Figure 5:**
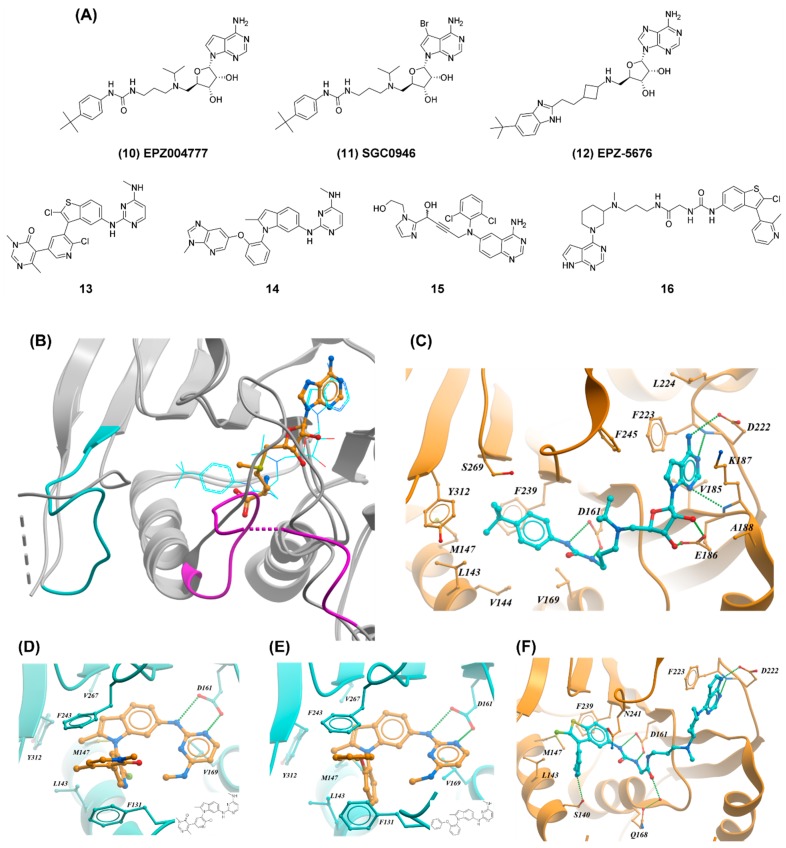
SAM-competitive inhibition of DOT1L. (**A**) Representative DOT1L inhibitors. (**B**) Crystal structure of DOT1L in complex with SAM (PDB ID: 3QOW) and EPZ004777 (PDB ID: 4EKI). For clarity, the structure of the cofactor is shown as orange balls and sticks, and EPZ004777 as cyan trace. The structure of both proteins is shown as grey ribbons. The activation and substrate-binding loops are colored in magenta and cyan, respectively in the EPZ004777 complex. (**C**) Crystal structure of DOT1L in complex with EPZ004777 (PDB ID: 4EKI). For clarity the activation and substrate-binding loops were hidden. (**D**) Crystal structure of a close analog of 13 in complex with DOT1L (PDB ID: 5DSX). (**E**) Crystal structure of a close analog of 14 in complex with DOT1L (PDB ID: 5DT2). (**F**) Crystal structure of DOT1L (PDB ID: 5MW4) in complex with 16. Hydrogen bonds are shown as green dotted lines.

The analysis of the reaction mechanism guided the design of the first DOT1L inhibitors by Epizyme [55,57]. Reducing the polar surface area (PSA) and replacing the homocysteine moiety with a phenyl-urea linker in an attempt to grow the inhibitor towards the lysine binding pocket led to the identification EPZ004777 (compound 10; *K_i_* = 0.3 nM) [55] (Figure 5A). EPZ004777 also inhibited H3K79 methylation in cells (IC_50_ = 5 nM) and selectively killed MLL-rearranged leukemia cells. Remarkably, this compound displayed a selectivity of >1000-fold for DOT1L over a subset of nine methyltransferases.

Kinetic studies using SPR showed that the improvement in target affinity of this series was driven mainly by a reduction in the dissociation rate constant (*k*_off_) resulting in a marked increase in residence time (τ) [57]. On the other hand, the association rate constant (*k*_on_) remained practically constant. These data suggested a conformational change of DOT1L that allowed binding of the more potent compounds.

Crystal structures of DOT1L in complex with EPZ004777 and analog SGC0946 demonstrated binding at the cofactor site and revealed a large structural remodeling of the activation and substrate-binding loops (residues 122-140 and 301–312, respectively) to accommodate the tert-butyl phenyl moiety of the inhibitor. This helped rationalize how these large and hydrophobic molecules could exploit the comparatively small and polar SAM binding pocket (Figure 4B,C) [56,57]. High affinity and extended residence time are directly associated with these conformational changes that dramatically increase the volume and hydrophobicity of the active site.

Following the observation that the N7 position of the adenine ring is surrounded with hydrophobic residues (F223, F245, and V249), Yu et al. designed and synthesized SGC0946 (11) (Figure 5A) [56]. This compound differed from EPZ004777 only by a bromine at position 7 of the adenine ring, which resulted in increased potency biochemically (*K*_D_ = 0.06 nM versus 0.25 nM) and in cells (IC_50_ = 9 nM versus 84 nM). Efforts to improve the pharmacokinetic properties of EPZ004777 led to the identification of EPZ-5676 (12), the first DOT1L clinical candidate (Figure 5A) [13]. This inhibitor is more potent and has better drug-like properties than previous compounds. EPZ-5676 inhibits the enzymatic activity of DOT1L with a *K*_i_ ≤ 0.08 nM, has a long residence time (>24 h) and >37,000-fold selectivity against a panel of 15 PMTs. EPZ-5676 is a potent inhibitor of H3K79 dimethylation with an IC_50_ value of 3 nM and 5 nM in MV4-11 and HL60 cell lines, respectively. This compound potently inhibits the expression of MLL-fusion target genes HOXA9 and MEIS1, selectively inhibits the proliferation of MLL-rearranged leukemia cells (IC_50_ = 3.5 nM), and causes complete and sustained regression in a rat xenograft model of MLL-rearranged leukemia.

Attempts to develop SAM-competitive DOT1L inhibitors structurally unrelated to the cofactor have been reported. In a high-throughput screening campaign, Chen et al. identified a low micromolar inhibitor [54]. A crystal structure showed that the compound induces a conformational rearrangement of the activation loop, opening-up a hydrophobic pocket adjacent to the SAM-binding site, which is occluded in the cofactor-bound structure. Several rounds of structure-based optimization led to 13 and 14 (Figure 5A). Both compounds are potent inhibitors of DOT1L with IC_50_ values of 1.4 and 0.4 nM, respectively, inhibited the proliferation of MLL-rearranged leukemia cell line MV4-11 (IC_50_ = 85 and 128 nM, respectively), and were selective against a panel of 22 PMTs. The crystal structure of DOT1L in complex with close analogs of 13 and 14 (Figure 5D,E) revealed that the diaryl core of both inhibitors fits tightly into the hydrophobic pocket formed by residues L143, M147, F239, and Y312.

DOT1L fragment screening was also successful. In two recent publications, researchers from Novartis described the discovery of two series of low nanomolar inhibitors [58,59]. In the first instance, Scheufler et al. identified a weak fragment hit using surface plasmon resonance (SPR). Structural information guided medicinal chemistry efforts to improve the potency and ligand efficiency (LE) of the fragment, which resulted in the discovery of 15 as a potent DOT1L inhibitor with an IC_50_ value of 14 nM (Figure 5A). The selectivity and cellular activity of 15 and other compounds from this series were not reported. The crystal structure of 15 in complex with DOT1L was not available, but a model of a close analog revealed that the compound does not bind in the SAM-binding site but occupies instead a pocket adjacent to the cofactor site [59]. Inhibitor binding induces a reorganization of the activation loop, opening a hydrophobic cavity formed by M147, F243, F239, and Y312 which is occupied by the 2,6-dichlorophenyl moiety of the inhibitor.

In the second publication, Mobitz et al. used a combination of fragment-based screen by nuclear magnetic resonance (NMR), virtual screening, and fragment linking approaches leading to the identification of 16, a highly potent DOT1L inhibitor with an IC_50_ < 0.1 nM and a *K*_i_ of 2 pM (Figure 5A) [58]. Of note, 16 performed equally or better than EPZ-5676 in cellular assays. The compound showed high cellular potency and no inhibition against a set of 22 PKMTs and PRMTs when tested up to 50 μM. The crystal structure of 16 in complex with DOT1L revealed that the pyrrolopyrimidine ring binds at the adenosine pocket of the protein while the benzothiophene moiety binds at the same hydrophobic pocket as the previously reported inhibitors from the same group [54,59] (Figure 5F).

### 2.4. EZH2

Polycomb repressive complex 2 (PRC2) silences gene expression through trimethylation of K27 of histone H3 (H3K27me3) [60]. PRC2 consists of four core subunits: EZH2 (the catalytic subunit), EED, SUZ12, and RBBP4 [61]. Aberrant PRC2 activity, sometimes caused by EZH2 mutations, is frequently associated with poor prognosis in hematological, lung, prostate, and breast cancer [62], and several small molecule inhibitors of EZH2 have recently been developed, including some currently evaluated in clinical trials.

**Figure 6:**
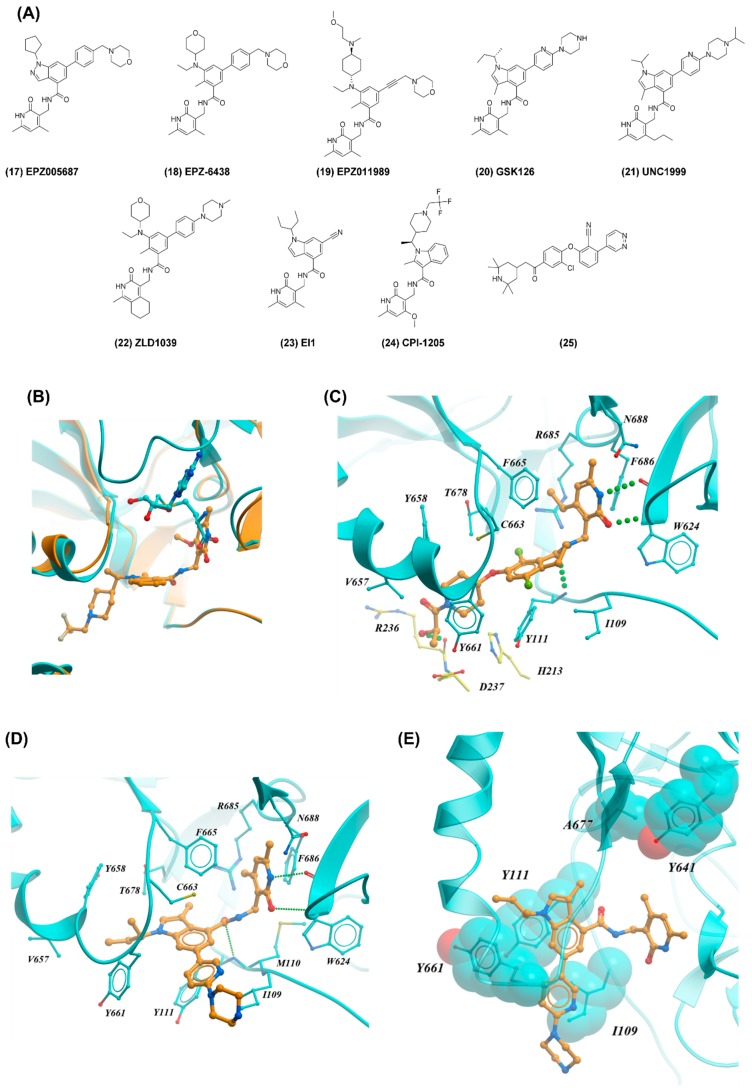
SAM-competitive inhibitors of EZH2. (**A**) Representative EZH2 inhibitors. (**B**) Crystal structure of PRC2 in complex with a close analog of CPI-1205 (orange, PDB ID: 5LS6). The structure of EZH2 in complex with SAH (PDB ID: 5HYN) is shown as cyan. (**C**) PRC2 crystal structure in complex with a lactam compound (PDB ID: 5IJ7). (**D**) Crystal structure of PRC2 in complex with GSK126 (PDB ID: 5WG6). Hydrogen bonds are shown as green dotted lines. (**E**) Crystal structure of PRC2-EZH2 in complex with GSK126 (PDB ID: 5WG6). The residues that are mutated in tumors (Y641, A677: oncogenic mutants; I109, Y111, Y661: drug resistance mutants) are shown as spheres.

The first reported potent and selective compound, EPZ005687 (17), inhibited EZH2 with an IC_50_ of 54 nM and was more than 500-fold selective over other methyltransferases except EZH1 (50-fold selectivity) (Figure 6A) [14]. The compound was also active against oncogenic forms of EZH2 mutated at Y641 and A677.

The same Epizyme team later reported the discovery of EPZ-6438 (18), and EPZ011989 (19) (Figure 5A) with improved pharmacokinetic properties compared to EPZ005687 [63]. EPZ-6438 was a potent inhibitor of wild-type EZH2 with a *K*_i_ of 2.5 nM and also inhibited EZH2 Y641 (F, C, H, N, S) and A677G mutants. It was completely selective for EZH2 against a panel of 14 methyltransferases and about 35-fold selective over EZH1. EPZ-6438 reduced the levels of mono−, di−, and trimethylation marks on H3K27 but no other histone marks. After 14 days of treatment, EPZ-6438 halted the antiproliferation of SMARCB1-deficient malignant rhabdoid tumors cells (G401) with nanomolar potency. Further studies revealed that EPZ-6438 induced complete tumor regression in G401 xenografts mice. Finally, EPZ-6438 is currently being evaluated in phase II clinical trials for the treatment of non-Hodgkin lymphoma (NHL) (Clinicaltrials.gov ID: NCT01897571).

In parallel, McCabe et al. identified GSK126 (20) (*K*_i_ = 0.5−3 nM) from the optimization of an HTS hit (Figure 5A) [15]. The compound showed remarkable selectivity (>1000-fold) for EZH2 over 20 other MTs, and was more than 150-fold selective for EZH2 over EZH1. Wild type DLBCL cell lines were less sensitive to GSK126 than the ones containing either Y641N, Y641F, or A677G mutations. Intraperitoneal administration of GSK126 induced reduction in tumor volume and increased survival in a mouse tumor xenograft model. GSK126 progressed to phase 1 clinical trials for the treatment of several tumors (Clinicaltrials.gov ID: NCT02082977) but was terminated as the maximal dose and schedule showed insufficient evidence of clinical activity.

Strikingly, the Epizyme and GSK ligands shared an amide of an aminomethylpyridone as a central core, and additional EZH2 inhibitors based on this scaffold were rapidly reported by other groups (compounds 21–24) (Clinicaltrials.gov ID: NCT02395601) [64,65,66,67]. Compound 25 is a potent EZH2 inhibitor diverging from the previous series [68]. Compound 25 inhibited wild-type EZH2, Y641N mutant, and EZH1 with IC_50_ values of 32, 197, and 213 nM, respectively, and displayed excellent selectivity. However, compounds with this scaffold have not demonstrated potency equivalent to pyridone-containing inhibitors and in vivo activity was not reported.

The crystal structure of PRC2 in complex with a close analog of CPI-1205 revealed that the compound occupies a pocket distinct from the cofactor binding site, at the interface of the EZH2 SET domain, the SAL region of the EZH2 N-terminus, and EED, an obligatory component of PRC2 (Figure 6B) [67]. Although the pyridone group of the inhibitor only partially overlaps with the carboxylic acid moiety of the cofactor, this limited steric clash is sufficient to prevent SAM binding, resulting in a SAM-competitive mechanism of inhibition. Most of the potency of the inhibitor relies on the rest of the molecule that extends away from the cofactor and binds in a juxtaposed pocket (Figure 6B).

The structures of PRC2 in complex with a lactam inhibitor discovered at Pfizer, and with GSK126 show a similar mechanism of action, where the conserved pyridone group overlaps minimally, but sufficiently with the cofactor binding pose (Figure 6C,D). Oncogenic mutations at Y641 and A677 are located ~7 Å away and have little impact on the activity of these inhibitors (Figure 6E). On the other hand, resistance mutations at Y661, Y111, and I109 led to significant loss in inhibitor potency [69]. Y661 and Y111 are in contact with each other and form a closed hydrophobic cavity that forms part of the pyridone binding site exploited by all catalytic EZH2 inhibitors (Figure 6E). Similarly, the side chain of I109 makes hydrophobic interactions with inhibitors. Importantly, all three mutations are remote from the cofactor binding pocket and have therefore no impact on cofactor binding. This underscores the fact that cofactor competitors that are not (or minimally) exploiting the SAM binding pocket are liable to mutations that affect inhibitor but not cofactor binding. In this regard, novel PRC2 inhibitors targeting a site of EED that is critical for methyltransferase activity seem to be less susceptible to resistance mutations [70,71].

## 3. Exploiting the Bound Cofactor

**Figure 7:**
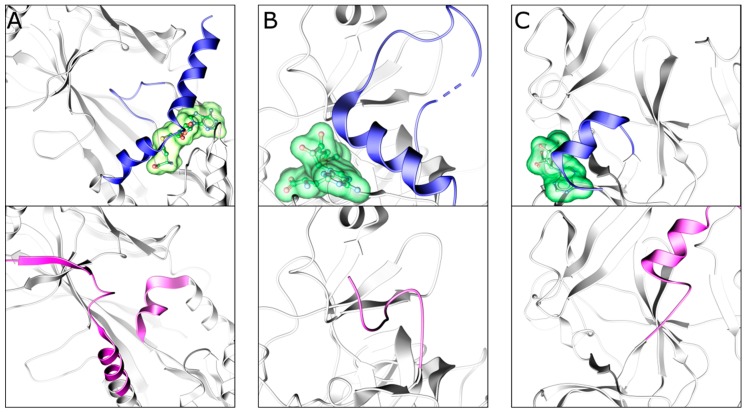
Cofactor binding induces structural rearrangements in methyltransferase domains. Cartoon representations highlighting the mobile structural elements of cofactor-bound (top, blue) and unbound (bottom, purple) conformations for (**A**) PRMT4, (**B**) SETD7, and (**C**) PRDM9. PDB IDs are 3B3F, 3B3J, 1N6C, 1H3I, 4C1Q, and 4IJD, respectively.

PMT substrate binding pockets are structurally more diverse and less polar than the SAM-binding pockets, and several chemical probes and compounds in clinical trials compete with the substrate rather than SAM. Interestingly, the presence of SAM or the reaction byproduct S-adenosylhomocysteine (SAH) can be required for substrate competitive inhibitors to bind their target enzymes, either via direct interactions between the cofactor and the inhibitor, or via cofactor-dependent allosteric stabilization of the substrate binding pocket. This structural plasticity is observed both in Class I and SET domain PMTs. For instance, cofactor binding can impose structural rigidity within the disordered substrate binding pocket of Rossmann proteins, as observed in the X-ray crystal structures of the SAM-bound and apo forms of PRMT4, which revealed a disorder-to-order transition of the substrate binding site induced by SAM [72,73] (Figure 7A). The post-SET domain of lysine methyltransferases can also require cofactor binding to properly fold and generate a substrate binding groove lined by the I-SET and post-SET domains [1,74]. For example, comparing the apo and SAM-bound forms of SETD7 reveals that the cofactor stabilizes residues along the substrate binding pocket [75,76] (Figure 7B). Moreover, a comparison of the human apo-PRDM9 and mouse holo-PRDM9 complexes clearly demonstrated the structural contributions of SAH to the catalytically competent conformation of PRDM9 [77] (Figure 7C).

**Figure 8:**
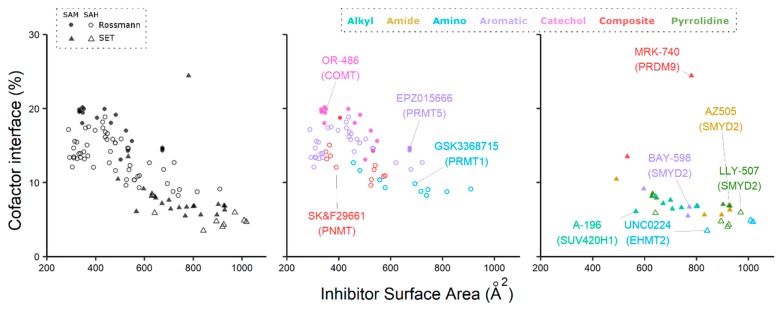
SAM or SAH interface bound by methyltransferase inhibitors. Percentage of the inhibitor’s interface interacting with SAM/SAH rather than with the protein (left panel). Inhibitors of Rossmann-fold (middle panel) and SET-fold (right panel) methyltransferases. Color-coding indicates inhibitor chemistry involved in SAM and SAH binding. Exemplary methyltransferase inhibitors are labeled with their target indicated in parentheses.

To illustrate the role of the methyltransferase cofactor for binding of substrate competitive inhibitors, we conducted a survey of all 102 methyltransferase structures where a small molecule inhibitor bound within <4 Å of either SAM or SAH. To examine cofactor contributions to inhibitor binding, we measured the contacting surface areas of each inhibitor with SAM/SAH and with the target protein and calculated the percentage of the interface formed with the cofactor (Figure 8). Cofactor-inhibitor interfaces can represent up to 20% of the total contact area of the inhibitor in Rossmann fold enzymes, while this percentage is generally lower in SET-domain PMTs, with the exception of PRMD9, where 24% of the inhibitor interface is with SAM. A detailed inspection of these structures revealed a diverse collection of inhibitor functional groups that interact with the cofactor (Figure 8, middle and right panels). In the following sections, we review the structural chemistry of substrate competing inhibitors that depend on the presence of the cofactor for binding.

### 3.1. PRMT5

**Figure 9:**
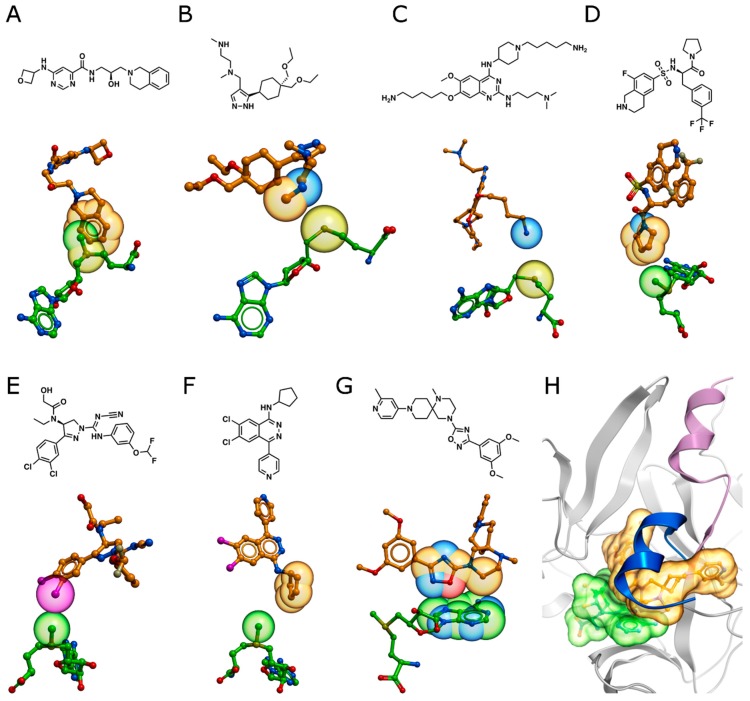
Substrate competitive methyltransferase inhibitors interact with SAM and SAH. Stick representations of inhibitor-cofactor pairs with spheres indicating van der Waals radii of interacting atoms for (**A**) EPZ015666-SAM (PRMT5, 4X61), (**B**) GSK3368715-SAH (PRMT1, 6NT2), (**C**) E72-SAH (EHMT1, 3MO5), (**D**) PFI-2-SAM (SETD7, 4JLG), (**E**) BAY-598-SAM (SMYD2, 5ARG), (**F**) A-196-SAM (SUV420H1, 5CPR), and (**G**) MRK-740-SAM (PRDM9, 6NM4). (**H**) MRK-740 locks the PRDM9 post-SET helix in an “open”, inactive confirmation (purple helix) and blocks the “closed”, active confirmation (blue helix, 4C1Q).

Substrate competitive Rossmann methyltransferase inhibitors have successfully employed aromatic, amide, and composite moieties to promote binding by forming stabilizing interactions with SAM and/or SAH (Figure 8). These compounds can possess high affinity for their target enzyme, such as the substrate competitive, SAM-dependent PRMT5 inhibitor, EPZ015666 (*K*_D_ < 1 nM) [16]. Interestingly, Chan-Penebre et al. could demonstrate that EPZ015666 possessed a much weaker affinity for PRMT5 when the SPR assays used to measure binding were performed in the presence of SAH (*K*_D_ = 171 nM) as opposed to SAM. Cocrystal structures of the SAM-PRMT5-EPZ015666 and SAH-PRMT5-EPZ015666 complexes illustrate how the phenyl ring of EPZ015666 forms an important cation-π interaction with the partially charged sulfonium methyl group of SAM that is absent in SAH (Figure 9A). By comparing the affinities of EPZ015666 to SAM− and SAH−bound PRMT5, the authors calculated that the cation−π interaction contributed~12.5 kJmol^−1^ of binding energy, demonstrating the specific importance of SAM for inhibition [16].

### 3.2. Type I PRMTs

Type I PRMTs are Rossmann-fold enzymes that catalyze the asymmetric di-methylation of a single nitrogen on the guanidinium moiety of arginine sidechains. Type I PRMT inhibitors that contain an aliphatic amine can function as unreactive surrogates for substrate mimetic inhibition. For example, GSK3368715 (EPZ019997) is a potent and bioactive pan-type I PRMT inhibitor currently in phase 1 clinic trial in patients with solid tumors and diffuse large B-cell lymphoma (Clinicaltrials.gov ID: NCT03666988). GSK3368715 is highly potent against all type I PRMT enzymes, with the exception of PRMT4 (IC_50_ values for PRMT1, PRMT3, PRMT4, PRMT6, and PRMT8 of 3.1, 48, 1148, 5.7, and 1.7 nM, respectively) [78]. Kinetic analysis of the inhibitory mechanism was performed by increasing either the substrate or SAM concentrations across enzymatic activity assays and indicated that GSK3368715 was a substrate competitive and SAM-dependent (also known as SAM-uncompetitive) inhibitor [78]. A cocrystal structure of the PRMT1-SAH-GSK3368715 complex demonstrated that the methylamino group of GSK3368715 projects through the substrate arginine site towards the sulfur atom of SAH (Figure 9B) [78]. Other type I PRMT inhibitors, such as TP-064 and MS023, also position an aliphatic amine within the arginine binding pocket, but instead exhibit SAM-independent (also known as SAM-non-competitive) modes of inhibition as determined by enzymatic activity assays [79,80]. Although an increase in SAM or peptide concentrations has no effect on the TP-064 catalytic IC_50_ value, TP-064 affinity for PRMT4 measured by surface plasmon resonance (SPR) revealed that the compound bound tightly to PRMT4 (*K_D_* = 7.1 ± 1.8 nM), but only in the presence of either SAM or SAH. Taken together, these findings exemplify the structural contribution of SAM or SAH towards the stabilization of the substrate pocket, which appears to be required for inhibition [73,80].

### 3.3. EHMT1/2 (G9a/GLP)

Aliphatic amines have also been employed by substrate mimetic inhibitors of SET-domain containing lysine methyltransferases. Chang et al. (2010) explored this idea by incorporating a lysine mimetic to a previously reported inhibitor (BIX-01294) of the histone H3 lysine 9 methyltransferases EHMT1 and EHMT2 (also known as GLP and G9a, respectively) [81]. Structural elucidation of EHMT1-SAH in complex with the modified inhibitor (E72) revealed that the primary amine is only 4.2 Å from the SAH sulfur atom and within the range of catalytic activity when EHMT1 is bound by SAM (Figure 9C). Indeed, the authors demonstrated that overnight incubation produced mono−, di−, and tri-methylated E72 and found that this slow methylation activity led to an improved IC_50_ value when compared to the inhibitory effect of the original BIX-01294 molecule [81].

Slow methylation of E72 highlights an interesting avenue for methyltransferase inhibition that exploits the natural function of SAM. In contrast to E72, inhibitor methylation was not reported for the EHMT1/2 inhibitor UNC0224, which possesses a terminal dimethyl-amino moiety [82]. A co-crystal structure of the EHMT2-SAH-UNC0224 complex revealed that the tertiary amine approaches the sulfur atom of SAH within a catalytically amenable distance similar to the structure of E72 bound to EHMT1 [82,83].

### 3.4. SETD7, SMYD2, and SUV420H1/2

Pyrrolidines are cyclic tertiary amines used in several unreactive substrate mimetic SET domain inhibitors that directly interact with the bound cofactor. For example, PFI-2 is a potent and cell-active SETD7 inhibitor that exhibits a substrate-competitive, SAM-uncompetitive mode of inhibition [84]. A cocrystal structure of SETD7 bound to SAM and PFI-2 demonstrated that the pyrrolidine group of the inhibitor extends through the substrate lysine channel and makes a hydrophobic interaction with the departing methyl group of SAM (Figure 9D). Importantly, affinity measurements by SPR confirmed that PFI-2 only binds to SETD7 in the presence of SAM [84].

BAY-598 is a peptide-competitive, SAM-uncompetitive inhibitor of SMYD2 that interacts with SAM via a chloro-substituted phenyl moiety [46]. A structure of the SMYD2-SAM-BAY-598 ternary complex revealed a direct contact between the 3-chloro substituent and the departing methyl group of SAM, resulting in both hydrophobic and weak electrostatic contributions to binding (Figure 9E). Hydrogen bonds can also form with the cofactor’s departing methyl group. For example, the cocrystal structure of the substrate-competitive, SAM-uncompetitive SMYD2 inhibitor AZ505 in complex with SMYD2 and SAM, showed that the ketone oxygen of the benzooxazinone moiety is 2.8 Å away from the sulfonium methyl group, which can act as a hydrogen-bond donor [47,85]. Stabilizing amide-sulfonium interactions have also been reported for other SMYD-family inhibitors such as EPZ030456 and an AZ505-analog called A-893 [86,87].

By forgoing the nitrogen atom present in the amino and pyrrolidine moieties of substrate mimetics, several inhibitors have used the enhanced aliphatic characteristics of cycloalkyl or simple alkyl moieties to bind within the substrate channel [49,88,89]. One example is the cyclopentane group on the SUV420H1/2 inhibitor A-196 [88]. Similar to the previously mentioned pyrrolidine inhibitor PFI-2, the cyclopentane group is positioned within 3.2 Å of the departing methyl group of SAM, making a hydrophobic interaction (Figure 9F). The authors used methyltransferase activity assays to assess the mechanism of inhibition and found that the IC_50_ value of A-196 remained constant with increased SAM concentrations, implying that A-196 is noncompetitive with the cofactor. Interestingly, isothermal titration calorimetry (ITC) affinity measurements demonstrated that the *K_D_* of A-196 decreases from 74.8 nM to 27.8 nM in the presence of SAM. The observed increased affinity of A-196 for SUV420H1/2 in the presence of SAM is attributed to the dual effect of interacting with SAM and stabilizing the substrate-binding fold of the protein, as observed in other SET domains and Rossmann domains [73,88,90].

### 3.5. PRDM9

Our structural survey of methyltransferase-cofactor-inhibitor complexes revealed that SET-domain inhibitors were generally larger than Rossmann methyltransferase inhibitors but possessed a smaller relative interface with either SAM or SAH compared to the protein (Figure 8). The only outlier to this trend was the PRDM9 inhibitor MRK-740, where SAM directly contributes to 24% of the total inhibitor binding interface. PRDM9 is one of seven PRDM family members with reported methyltransferase activity. PRDMs share the SET-domain fold but have only 20–30% amino acid sequence identity with other SET-domain enzymes [83]. MRK-740 is a PRDM9-specific, first-in-class PRDM inhibitor with a unique mode of binding compared to all SET-domain inhibitors [91]. Enzyme assays demonstrated that MRK-740 is a substrate-competitive, SAM-uncompetitive inhibitor, and a cocrystal structure of the PR-domain of PRDM9 in a ternary complex with SAM and MRK-740 revealed that MRK-740 makes multiple interactions with SAM and PRDM9 driven by aromatic and hydrophobic substituents on the inhibitor (Figure 9G). MRK-740 locks the enzyme into an inactive, “opened” conformation that prevents completion of the substrate pocket, while simultaneously occupying the lysine binding cavity (Figure 9H). The extensive interface between MRK-740 and SAM highlights essential structural contributions provided by the cofactor. Whether such an extensive inhibitor-cofactor interaction is only possible within the PRDM subfamily remains an open question.

## 4. Conclusions

PMTs have emerged as a promising target class in oncology and other disease areas. As the chemical coverage of this protein family is progressing, it is clear that SAM plays a central role in the mechanism of action of PMT inhibition. Here, we reviewed challenges associated with cofactor competition, and solutions revealed by high-quality cofactor competitors. We also highlighted how substrate competing compounds could favorably exploit the bound cofactor as an interaction hotspot. Not discussed here are other successful mechanisms of action, such as allosteric inhibition [92], or targeting of alternative subunits of methyltransferase complexes [70,71,93]. Finally, the possibility of targeting methyl-lysine binding or other domains often juxtaposed to the catalytic methyltransferase domain remains an underexplored avenue.
